# Genomic evolution of the SARS-CoV-2 Variants of Concern: COVID-19 pandemic waves in India

**DOI:** 10.17179/excli2023-6098

**Published:** 2023-06-01

**Authors:** Pooja Bhardwaj, Shailendra Kumar Mishra, Sthita Pragnya Behera, Kamran Zaman, Rajni Kant, Rajeev Singh

**Affiliations:** 1 Indian Council of Medical Research (ICMR) - Regional Medical Research Center Gorakhpur, BRD Medical College Campus, Gorakhpur-273013, U.P., India

**Keywords:** COVID-19, India, SARS-CoV-2, Spike protein, Variant of Concern

## Abstract

SARS-CoV-2 has mutated rapidly since its first case report in Wuhan, China, leading to the emergence of an indefinite number of variants. India has witnessed three waves of the COVID-19 pandemic. The country saw its first wave of SARS-CoV-2 illness from late January 2020 to February 2021. With a peak surge of cases in mid-September 2020, India recorded more than 11 million cases and a death toll of more than 0.165 million at this time. India faced a brutal second wave driven by the emergence of highly infectious SARS-CoV-2 variants B.1.617.2 (Delta variant) and the third wave with the leading cause of BA.2 (Omicron variant), which has led to an unprecedented rise in COVID-19 cases in the country. On September 14, 2022, India recorded a cumulative 44.51 million cases of COVID-19 with more than 0.528 million deaths. The discovery of common circulating mutants is facilitated by genome sequencing. The changes in the Spike surface glycoprotein recombinant binding domains served as the critical alterations, resulting in enhanced infectivity and transmissibility, with severe clinical effects. Further, the predominant mutation in the SARS-CoV-2 spike protein; the D614G strains served as a model for vaccine development. The mutation of the Wuhan strain to the Variant of Concern led to a significant increase in SARS-CoV-2 infections. In addition, there was a shift in the age group affected by SARS-CoV-2 variant infection. The current review summarized the COVID-19 pandemic's Variant of Concern and the advent of SARS-CoV-2 in India.

## Introduction

In late December 2019, the city of Wuhan, China, reported the appearance of a cluster of atypical pneumonia cases caused by a novel human coronavirus (Severe Acute Respiratory Syndrome-Coronavirus-2, SARS-CoV-2) (Hu et al., 2021[25]; Li et al., 2020[32]). On February 11, 2020, the World Health Organization (WHO) named the illness brought on by the SARS-CoV-2 virus as coronavirus disease-19 (COVID-19). The WHO designated COVID-19 as a pandemic on March 11, 2020, and SARS-CoV-2 as a public health emergency of international concern (PHEIC) on January 30, 2020 (Cucinotta and Vanelli, 2020[15]). As of January 27, 2023, the COVID-19 pandemic had brought in 752,517,552 confirmed cases of the disease, along with 6,804,491 fatalities (https://covid19.who.int/).

India documented its first case of SARS-CoV-2 in Kerala state on January 30, 2020, from an Indian medical student who returned from Wuhan, China on January 27, 2020 (Andrews et al., 2020[1]). Later, two new positive cases were detected; one patient returned from Vienna, the Capital of Austria, and the other from Dubai on March 2, 2020. Subsequently, the continuity of the SARS-CoV-2 infections has occurred persistently in seasonal, geographical, and vaccination-dependent manners with rising or declining trends of SARS-CoV-2 infection cases. As of September 14, 2022, the WHO documented over 44 million SARS-CoV-2 confirmed cases, and the death toll attributed to COVID-19 had reached 528,216 in India. Among Asian countries, India is on top of confirmed COVID-19 cases and has the second most confirmed cases across the globe after the United States of America. SARS-CoV-2 outbreaks caused by human-to-human transmission created a major public health issue. To enforce social distancing, a "Janata curfew" (14-hour lockdown) was implemented on March 22, 2020, by the Government of India (GoI). Further, GoI has decided to impose a 21-day (24 March - 14 April 2020) lockdown (Phase-I) across the country to restrict the movement of 1.3 billion population as a preventive measure against the COVID-19 pandemic (Kumar et al., 2020[29]). The lockdown period for phase II was prolonged by an extra 19 days (15 April to 3 May), for phase III by 14 days (4-17 May), and phase IV by 14 days due to the sudden rise in cases (18-31 May). As of May 4, 2020, the Ministry of Health & Family Welfare (MoHFW) had further designated at least 130 districts as "hotspot" or "red" zones, 284 as "orange" zones (with fewer cases of SARS-CoV-2), and 319 as "green" zones (with no cases of SARS-CoV-2). The Indian Council of Medical Research (ICMR) has taken a lead role in efforts to develop significant capacities for various aspects of COVID-19 management, such as T3; testing, tracking, and treatment.

Amid the lockdown, people faced enormous social, environmental, health, and economic challenges. The implementation of a countrywide lockdown led to the shutting down of factories and industries with millions of workers unemployed. These unemployed laborers return to their hometowns from metropolitan cities across India, which were earlier notified as the major hot spots of COVID-19 infections in the country.

**Figure 1:**
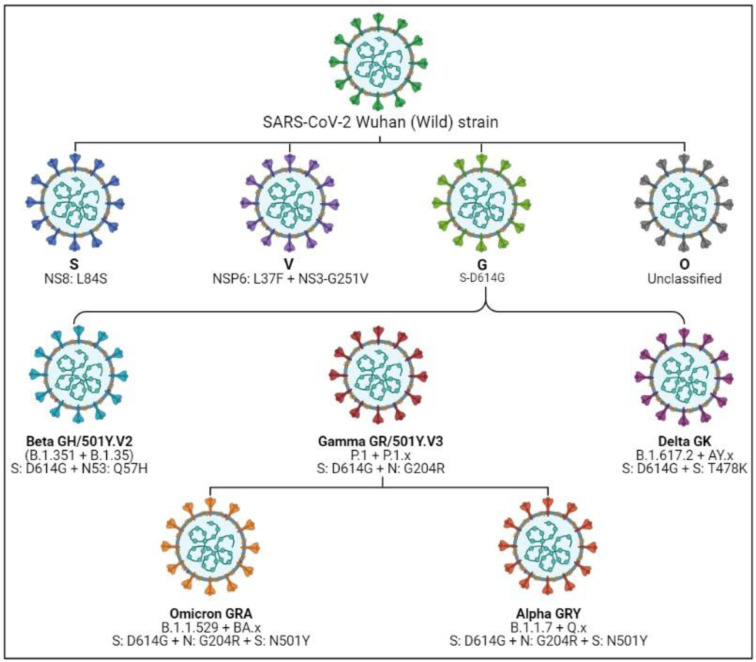
Trend of emerged SARS-CoV-2 GISAID clades (Circle) and their respective marker mutations (Box). Bold letters in the box represent the gene in which mutation is depicted.

**Figure 2:**
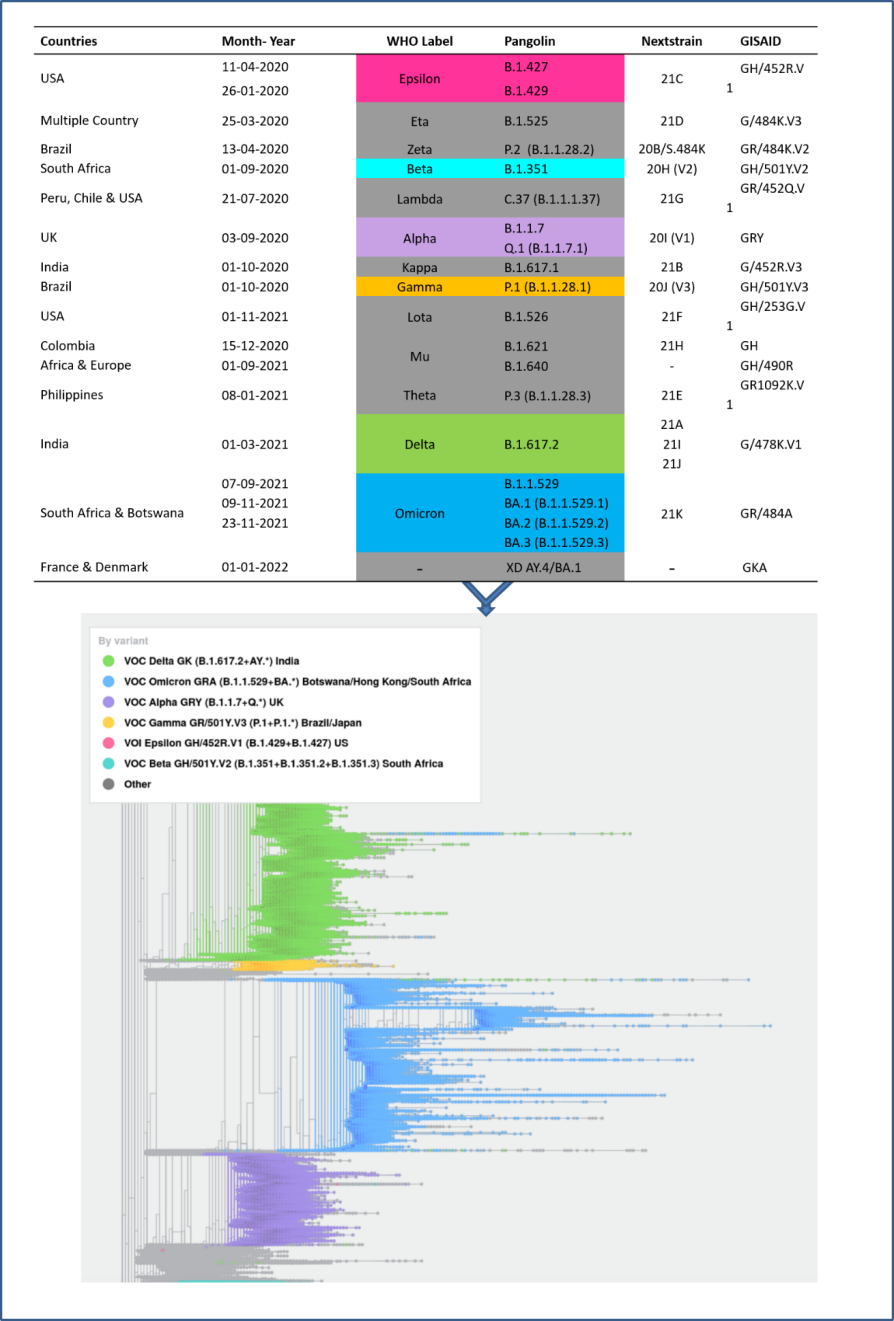
Different nomenclature systems used for classification of SARS-CoV-2 variants and their evolutionary relationship. The GISAID classification refers to key mutations observed during evolution, Nextstrain classification refers to the year and clades evolved and Phylogenetic assignment of named global outbreak lineage (PANGOLIN) classification followed Rambaut et al. (2020).

The Global Initiative on Sharing All Influenza Data (GISAID) introduced the nomenclature of SARS-CoV-2 to keep track of changes in the SARS-CoV-2 genome. According to GISAID nomenclature, the clades were designated according to the marker mutation. For instance, the G clades refer to the marker mutation in the S protein; S-D614G (an aspartic acid-to-glycine substitution at position 614 of the spike glycoprotein) is responsible for its rapid transmission and hence emerged as a major strain (Lauring and Hodcroft, 2021[31]). Likewise, in addition to D614G further addition of mutation in RBD of S protein as well as other structural proteins, split the G clade into GH (NS3-Q57H), GR (N-G204R), GK (S-T478K) and GV (S-A222V) which additionally contributed to its increased pathogenicity and immune escape properties. In late 2020, GR clade further diversified into GRY (Alpha) clade with potential mutation and deletion (Figure 1(Fig. 1)) (GISAID, 2021[21]). The introduction of mutations in the structural protein of SARS-CoV-2 enabled them to progressively prevail across the country. The SARS-CoV-2 GR clade has further evolved into the GRA clade (Omicron) with over 50 potential mutations and deletions. The different nomenclature system for SARS-CoV-2 variants including WHO Greek, Pangolin and Nextstrain is given in Figure 2(Fig. 2) (Reference in Figure 2: Rambaut et al., 2020[44]).

With the genome sequence of the SARS-CoV-2 virus becoming available in early January 2020, the alarming situation increased the urgency of developing a vaccine to mitigate the SARS-CoV-2 impact on public health, the economy and society. By the beginning of April 2020, Spike D614G mutation exhibited increasing in frequency across multiple geographic locations including India and G614 has since become a prevalent SARS-CoV-2 variant in the global outbreak (Korber et al., 2020[28]; Sarkale et al., 2020[46]). The dominant strain D614G was then utilized as a platform for the development of all the vaccine candidates (Kumar et al., 2020[29]). As of March 30, 2022, 153 vaccines were in clinical development and 196 were in pre-clinical development. The Drugs Controller General of India (DCGI) has granted an emergency use authorization (EUA) to COVAXIN, ZyCoV-D, and CORBEVAX for the vaccination of adolescents aged 15-17. As of January 12, 2022, nine COVID-19 vaccines have obtained Emergency Use Listing (EUL) from the WHO. Of them, BBV152/COVAXIN (BBV152), an indigenous vaccine for COVID-19 based on the whole inactivated virus was developed in India by a collaborative approach of the Indian Council of Medical Research (ICMR) - National Institute of Virology (NIV) and Bharat Biotech International Limited, India. 

Studies have demonstrated that mutations in the S-glycoprotein alter the adaptability and infectivity of SARS-CoV-2, but how the structural genes evolved during human-to-human transmission is not known (Andrews et al., 2020[1]; GISAID, 2021[21]; Lauring and Hodcroft, 2021[31]). The mutations in the SARS-CoV-2 may enable it to escape the immune system and alter its infectivity, reproducibility, and immune escaping properties. Based on these characteristic features, WHO has categorized such variants as Variants of Concern (VOC). This review describes, in brief, the SARS-CoV-2 evolution, its VOC and its introduction in India and its role in different waves of pandemics in India.

### SARS-CoV-2 evolution

The biggest subfamily of the Coronaviridea, known as Coronaviruses (CoVs), is composed of enclosed positive-stranded RNA viruses. Seven coronaviruses have been identified in the last 20 years, with SARS-CoV-2 becoming the eighth coronavirus to infect humans. SARS-CoV-2 was found to be a member of the Betacoronavirus genus, which also includes the coronaviruses MERS-CoV, SARS-CoV, SARS-related coronaviruses from bats (SARSr-CoV), and additional viruses that can infect both humans and a variety of animal species (HCoV-OC43, HCoV-HKU-1, HCoV-229E, and human species HCoV (Holmes et al., 2021[24]; Singh and Yi, 2021[50]; Tabibzadeh et al., 2021[53]). Early WGS (whole-genome sequencing) comparisons of viruses most closely related to SARS-CoV-2 revealed an impressive array of bat coronaviruses, including Bat SARSr-CoV RaTG13 (96.2 %) followed by Bat SARSr-CoV CoVZC45 (88.1 %) and SARSr-CoV CoVZXC21(88.0 %) (Zhou et al., 2020[62]). 

SARS-CoV-2 has the largest RNA genome (around 30 kb) in length, composed of open reading frames (ORFs) arranged as replicase and protease (1a-1b) and four major structural proteins, that include spike (S), envelope (E), membrane (M), and nucleocapsid (N) protein (Mariano et al., 2020[37]). Receptor-Binding Domain (RBD) is a critical region of the SARS-CoV-2 virus located on homotrimeric spike glycoprotein. Further, spike RBD of SARS-CoV-2 is essential for binding to the human cell receptor Angiotensin-Converting Enzyme 2 (ACE2) and invading the host cells (Lan et al., 2020[30]). SARS-CoV-2 carrying several amino acid changes in spike protein results in infectivity of the COVID-19 disease in humans and thus accumulating mutations over time has become a public health concern globally. According to previous studies, mutations are responsible for the rapid spread and evolution of SARS-CoV-2 (Phan, 2020[43]). 

The spike surface glycoprotein of SARS-CoV-2 is required for attachment of the virus to host cell surface receptors and promotes the fusion of the virus into cell membranes and hence facilitates virus penetration (V'kovski et al., 2021[57]). It is also a popular target for neutralising antibodies (Ju et al., 2020[26]). Further, the S protein recombinant binding domain (RBD) of SARS-CoV-2 binds to ACE2 with great affinity explaining its highest infection potential than SARS-CoV. This emphasizes the significance of structural protein changes, particularly the RBD of the S protein, which interacts with the ACE2 protein, during SARS-CoV-2 progression (He et al., 2020[22]). With each mutation in the structural gene of SARS-CoV-2, the evolved variants of SARS-CoV-2 may act differently and could become more lethal and leading to an increase in case fatalities. Mutations in the RBD of S surface glycoprotein of SARS-CoV-2 lead to conformational alterations, which most likely lead to antigenic shift. 

Under considerable selection pressure, insertion, deletion, antigenic shift, antigenic drift, or intragenic recombination may have played a critical role in the evolution of SARS-CoV-2. Although the Coronaviridae family has good replication fidelity, mutations are occurring at a faster rate than expected (Telenti et al., 2021[55]). Furthermore, it was discovered that RNA recombination events occurred frequently in coronaviruses. Antigenic drift can occur as SARS-CoV-2 S protein mutations accumulate (essential for host antibody recognition), allowing the virus to evade vaccinations and innate immune responses. It is conceivable that a protracted infection in an immunocompromised person will lead to the accumulation of mutations in the SARS-CoV-2 genome. Additionally, it is difficult to rule out the possibility of SARS-CoV-2 and other human coronavirus variants undergoing recombination processes. Hence, humans infected with multiple variants of SARS-CoV-2 at a time may serve as a reservoir for potent recombination events leading to antigenic shift and thus can allow the SARS-CoV-2 virus to invade multiple hosts at a time (Forni et al., 2017[20]). Some evidence for the positive selection of S-glycoproteins has recently been found (Zhan et al., 2020[61]). There is evidence that the continuous regions of S-glycoprotein and N-gene of SARS-CoV-2 are the result of intragenic recombination between RaTG13 and Guangdong (GD) Pangolin CoVs through convergent evolution (Makarenkov et al., 2021[35]). Tang et al. (2020[54]) demonstrated that natural selection, rather than recombination, induced alterations in the functional site in the S protein of SARS-CoV-2 (Tang et al., 2020[54]). Such studies emphasized the imperative requirement for further research that combine viral genomic data with coronavirus epidemiological investigations of the SARS-CoV-2 outbreak.

### SARS-CoV-2 variants 

SARS-CoV-2 variants were defined as genomes with single or more significant mutations that differed in antigenicity or virulence. The Centers for Disease Control (CDC) divided these variants into three groups based on the severity of the clinical consequences caused by the SARS-CoV-2 virus: "Variants of Interest (VOI)", "Variants of Concern (VOC)", and "Variants under surveillance" (VUM) (WHO, 2023[59]). A variant is VOI if it has mutations that create unique outbreak clusters and is extensively circulated. Whereas VOCs are hypothesized to be capable of rapid transmission, increased hospitalizations or fatalities, evading the body's immune response, altering clinical presentation, as well as reducing the efficacy of diagnostics, therapies, and immunizations. The list of currently designated VOC, VOI and VUM is given in Table 1(Tab. 1). Since, the first confirmed report of SARS-CoV-2, the world has encountered several variants of SARS-CoV-2, among which some variants were of low virulence and vanished with time. Wherein, some variants evolved during 2020 such as Alpha (B.1.1.7 and Q lineages), Beta (B.1.351 and descendent lineages), Gamma (P.1 and descendent lineages) and Delta (B.1.617.2 and AY lineages), and Omicron (B.1.1.529 and BA lineages) (evolved during 2021) were designated as VOCs because of their high virulence, rapid transmission, disease severity and breakthrough infections (Forni et al., 2017[20]; He et al., 2020[22]; Tang et al., 2020[54]; Telenti et al., 2021[55]). Clinical manifestations have led to the conclusion that infection from SARS-CoV-2 can vary from mild to severe, as asymptomatic cases have also been reported (Deval et al., 2022[16]; WHO, 2023[59]; Tang et al., 2020[54]). 

#### SARS-CoV-2 Alpha Variants: First wave of infections

The first case of SARS-CoV-2 in India was documented in January 2020 (Andrews et al., 2020[1]) and the first wave continued for far more than a year. The first wave of the pandemic showed a steep rise in cases during September (90,000 cases/day) (Chakraborty et al., 2022[10]). The mutated variant of the Wuhan strain with D614G mutation was prevalent worldwide. The substitution of G for D in the Spike protein induces a relaxation of the strained hydrogen bond, increasing the probability of binding events with the host ACE2 receptor and so increasing the virus's transmissibility and infectivity (Thakur et al., 2022[56]). 

**Figure 3:**
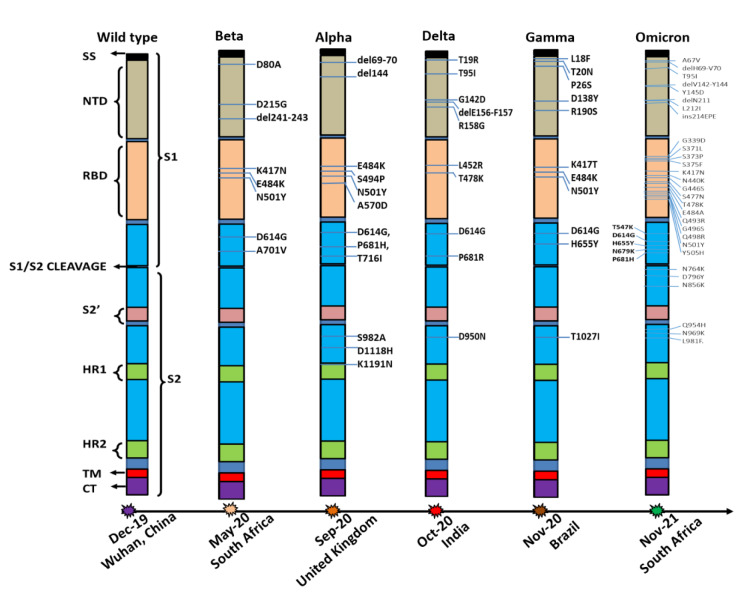
Timelines for the Variant of Concerns evolved during the COVID-19 pandemic with their respective country reported first. Bar graphs represent key mutations in spike glycoprotein with respect to wild type. Bar graph of wild type showed S1 fragment containing SS- signal sequence, NTD- N-terminal domain, RBD- Receptor-binding domain, S1/S2 cleavage site and S2 fragment with Two HR-Heptad repeats (HR1 & HR2), TM-Transmembrane anchor, CT- C-terminal

The SARS-CoV-2 Alpha VOC, which is from the B.1.1.7 pangolin lineage, was discovered for the first time in the UK in September 2020. At the end of the first wave and the start of the second wave of pandemics in India, the Alpha strain appeared to be dominant (Dhar et al., 2021[17]). The three most important mutations in this strain's RBD area are E484K, S494P, and N501Y. Other significant S-glycoprotein mutations besides RBD include the following: 69del, 70del, D614G, 144del, A570D, S982A, P681H, D1118H, T716I, and K1191N (Figure 3(Fig. 3)). During the first wave of pandemic population the age group of 35.1±15.9 were majorly affected (Dhar et al., 2021[17]; Thakur et al., 2022[56]). With its three clinically significant mutations, N501Y (S protein interaction with ACE2), double deletion at 69/70 (conformational modifications to S protein), and P681H (S1/S2 cleavage site alteration), this variant showed increased severity and case fatalities (Dhar et al., 2021[17]; Reddy et al., 2021[45]; Sarkar et al., 2021[47]). Furthermore, significant mortality from SARS-CoV-2 infections during the first wave could be related to a lack of hospitality and testing laboratories, a lack of evidence on viral pathogenesis and transmission, vaccine shortages, and medicine shortages (Thakur et al., 2022[56]). The alpha variant has been shifted from the VOC to the VUM category as of September 21, 2021 (CDC, 2023[8]). 

#### SARS-CoV-2 Beta and Gamma Variants

The SARS-CoV-2 Beta and Gamma variants of the B.1.351 and P.1 Pangolin lineages, as well as their descendent lineages, were initially identified in May 2020 and November 2020, respectively, in South Africa and Brazil. K417N, E484K, and N501Y are major mutations in the RBD region of Beta variants, while D80A, D215G, 241del, 242del, 243del, D614G, and A701V are modifications in S protein (Figure 3(Fig. 3)). The Gamma VOCs include mutations in RBD such as K417T, E484K, and N501Y, as well as T20N, R190S, D614G, P26S, D138Y, H655Y, L18F, and T1027I in S proteins other than RBD (Figure 3(Fig. 3)). Despite the fact that both were categorized as VOCs, India was least affected by these two variants. These Beta and Gamma variants have also been shifted from the VOC to the VUM category as of September 21, 2021. 

#### SARS-CoV-2 Delta Variant: Second wave of infections

After surviving the first wave of the SARS-CoV-2 infections relatively undamaged, India was hit by a vicious second wave in March 2021, accounting for over half of all global occurrences by the first week of May 2021 (Kar et al., 2021[27]). Soon the Delta variant took over the charge of the second wave of the COVID-19 pandemic from Alpha variants and resulted in overburdening of the healthcare system. A sudden rise in cases during this phase leads to a shortage of medical oxygen, hospital beds, and other necessities for COVID-19 patients. The SARS-CoV-2 Delta variants, which first appeared in India in October 2020, correspond to the Pangolin lineages B.1.617.2 and AY. The Delta variants appeared with vital mutations in RBD including L452R and T478K, whereas other mutations in S proteins include T19R, (V70F), T95I, G142D, E156-, F157-, R158G, (A222V), (W258L), (K417N), D614G, P681R, D950N (Figure 3(Fig. 3)) (Vasireddy et al., 2021[58]). This variant has proliferated throughout India. During the second wave, the age group affected was 46.1±16.8 (Dhar et al., 2021[17]; Thakur et al., 2022[56]). Further, the Delta variant was the most common source of SARS-CoV-2 behind COVID-19 infection in Indian children (<18 years), followed by Kappa, Alpha, and B.1.36. variant during the second wave of the pandemic in the country (Yadav et al., 2022[60]).

The structural analysis showed that RBD mutations L452R and T478K in the S-glycoprotein of the delta variant can promote ACE2 binding, while P681R in the furin cleavage region can increase the rate of S1-S2 cleavage, leading to increased transmissibility (Cherian et al., 2021[12]). The spike's N-terminal domain (NTD) mutations (T19R, R158G, del156-157) in the SARS-CoV-2 Delta variant occur on the major monoclonal antibody binding site, suggesting that this strain is more efficient at evading antibody recognition (Hoffmann et al., 2021[23]). The Delta VOC surpassed its predecessors by incorporating modifications that increased replication, immune evasion, and host receptor avidity, improving transmissibility, reinfection, and vaccination breakthrough (Dhar et al., 2021[17]). Delta variant breakthrough re-infect-ion in a patient with breakthrough infection of Alpha variant, complete vaccination showed the severity of disease caused by this variant (Shastri et al., 2021). The first wave, according to the researchers, was more lethal than most people realize, but the second wave was more terrible to experience since it was compacted into a few weeks, overburdening hospitals and oxygen supplies (Dyer, 2021[19]).

#### SARS-CoV-2 Omicron Variants: Third wave of infections

The Omicron variant is currently the most predominant SARS-CoV-2 variant that largely replaced previous circulating strains such as alpha, beta, gamma and delta. The Omicron variant (BA.1) appears to have emerged in November 2021 from South Africa and has expanded substantially since then to several other countries across the globe. In January 2022, the original Omicron strain BA.1 was largely replaced by BA.2; and other subvariants such as BA.3, BA.4, and BA.5 have subsequently emerged. Omicron variants with over 50 mutations belong to the B.1.1.529 and BA pangolin lineages. Among the 50 mutations of the Omicron genome, 15 mutations are located in the RBD leading to the highest infectivity and immune escape. The key mutations in S protein of Omicron include A67V, del69-70, T95I, del142-144, Y145D, del211, L212I, ins214EPE, G339D, S371L, S373P, S375F, K417N, N440K, G446S, S477N, T478K, E484A, Q493R, G496S, Q498R, N501Y, Y505H, T547K, D614G, H655Y, N679K, P681H, N764K, D796Y, N856K, Q954H, N969K, L981F (Figure 3(Fig. 3)). Omicron complex have continued to circulate globally, accounting for >98 % of viral sequences shared on GISAID after February 2022.

As of December 2, 2021, the Omicron variant (B.1.1.529) was confirmed in two cases with international travel history from Southern India (Scully, 2021[48]). Since then, there has been an increase in the number of Omicron variant infection cases across the country, indicating the potential of super-spreadability. The third wave of SARS-CoV-2 illnesses in India was brought on by the VOC Omicron. The third wave of infections stroked India amid December 2021 with its peak in February 2022. Studies with Omicron RBD NAbs revealed that mutations in the Omicron variant allowed it to escape NAbs from epitope groups A-F, specifically A-D (Cao et al., 2022[6]). Preliminary findings indicate a higher risk of reinfection and vaccination failure in COVID-19 recovered patients. Omicron's total genetic alterations conferred increased contagiousness, immunological escape from the vaccine and immunotherapy-based protection, and diagnostic hindrance (S-gene dropout) (Cao et al., 2022[6]; Mohapatra et al., 2022[40]; Patil, 2022[41]). Fortunately, the Omicron variant is highly transmissible but not lethal, as disease severity, hospitalization, and respiratory support have decreased (Christensen et al., 2022[13]). Omicron has been a hidden blessing that will help bring the COVID era to an end. Mother Nature will keep immunising humans with Omicron and future variants. When Omicron emerges, human cellular intelligence allows it to weaken the SARS-CoV-2 (Chaturvedi and Badwe, 2021[11]). 

SARS-CoV-2 has shown an unrivalled ability to evolve and take over the charges from contained variants in a shorter period before building up selective pressure (Telenti et al., 2021[55]). Recently, the U.K. Health Security Agency (UKHSA) has reported three recombinants of the SARS-CoV-2 variant, known as XF, XE and XD. Of these, XD and XF are recombinants of Delta and Omicron BA.1, while XE belongs to recombinants of both sub-variants BA.1 and BA.2 of super spreader Omicron. The WHO has flagged the emergence of XE recombinant with a higher rate of transmission of about 10 % as compared to the Omicron BA.2 variant. India reported the first case of XE variant of the SARS-CoV-2 virus in a 50-year-old fully vaccinated South African female from Mumbai on April 6, 2022. As Omicron cases have spread globally, researchers at the IHU Institute for Mediterranean Infection in France reported another COVID-19 variant called 'IHU' (also known as B.1.640.1) had more mutations (46 mutations and 37 deletions), including 14 amino acid substitutions and 9 deletions are sited in the spike protein (Colson et al., 2022[14]). The incidence of the IHU variant has been outnumbered by the superspreader Omicron variant, rendering most people immune to the virus and thus, IHU did not impact significantly as they may already have developed immunity towards the IHU variant. Hence, the number of mutations and deletions is unrelated to infectiousness and pathogenicity, but rather to variations in the receptor binding domain of spike protein.

##### Omicron subvariants

Since emerging in November 2021, the highly transmissible SARS-CoV-2 omicron strain has splintered into an expansive array of subvariants that are driving new spikes in Covid-19 cases around the world (Petersen et al., 2022[42]). The vast genetic diversity of Omicron sublineages displays similar clinical outcomes but different immune escape potentials. The immune evasion potential of Omicron variants is strongly influenced by the regional immune landscape. As per the WHO, as of January 23, 2023, four subvariants under monitoring are BF.7 (BA.5 + R346T mutation in spike), BQ.1* (and BQ.1.1, with BA.5 + R346T, K444T, N460K mutations in spike), BA.2.75* (including BA.2.75.2 and CH.1.1), and XBB* (including XBB.1.5) at the global level. The XBB and BQ.1.1 exhibited the strongest resistance to monoclonal antibodies (mAbs) that target RBD and enhanced ACE2-binding affinity (Cao et al., 2023[5]; Mohapatra et al., 2022[39]). According to the data of the Indian SARS-CoV-2 Consortium on Genomics (INSACOG), the recent rise in infections in India demonstrates that most cases were due to XBB (BA.2.10) strain (63.2 %) followed by BA.2.75 and BA.2.10 in a lesser extent. Some occurrence of BF.7 sub-lineage has also been seen in the Eastern and Northern parts of India.

## Vaccine Effectiveness in Preventing SARS-CoV-2 Infection

The search to develop a vaccine has begun with the availability of the genome sequence in early 2020. The first mass vaccination program was started in December 2020 and the number of vaccination doses administered is updated regularly on the WHO COVID-19 dashboard. As of 12 January 2022, nine vaccines have obtained EUL (Emergency Use Listing) by WHO. The position D614 had identified main target by the researchers for the development of a vaccine against SARS-CoV-2 that can neutralize infection is the spike (S) glycoprotein (Singh et al., 2022[51]). Therefore, research institutes and industry have collaborated to develop vaccines based on S-glycoprotein and its antigenic domains and epitopes that have been shown to be effective against the D614G form of the virus (Dong et al., 2021[18]). The 'S' glycoprotein-based vaccine has been shown to be effective in generating neutralizing antibodies against the Wild type Wuhan strain. SARS-CoV-2 variants carrying the D614G did not demonstrate major resistance to either vaccines or immune escape and are therefore not considered an escape mutation, hence the vaccine developed by targeting D614 was effective against D614G form of SARS-CoV-2 (Bano et al., 2022[2]).

In India, through the joint efforts of the Indian Council of Medical Research and Bharat Biotech Ltd. developed the first approved indigenous vaccine 'Covaxin' protected against SARS-CoV-2 infection. However, the biggest hurdle to vaccine efficacy is that the virus is a positive ssRNA, which is more susceptible to mutations, and this property of recurrent mutations puts the vaccine's effectiveness in question. Under constant selection pressure, SARS-CoV-2 continued to mutate and evolve, and most mutations in S-glycoprotein were associated with transmissibility, infectivity, and the level of resistance to antibody-mediated neutralization of the virus (Malik et al., 2022[36]; Singh et al., 2021[52]). The studies conducted to evaluate the effectiveness of vaccine in preventing SARS-CoV-2 breakthrough infection had demonstrated that one in five fully vaccinated individuals was estimated to have SARS CoV-2 infection. Indeed, large scale vaccination reduces lengths of hospital stay and likelihood of serious illness (Behera et al., 2022[3]; Singh et al., 2021[49]).

Indian isolates of SARS-CoV-2 exhibited mutations in different genomic regions, with substitutions in spike protein notably accounting for the potential impact on various functions, including antigenic diversity and immune escape of SARS-CoV-2 (Limaye et al., 2021[33]). The Delta variant of SARS-CoV-2 was first spotted in India, leading to the deadly third wave in the country and rapidly replaced other existing variants around the globe. This virulent strain thus challenged the efficacy of the vaccines and therapeutics developed, led to the breaching of the immune system barrier and led to infection and reinfection in fully and partially vaccinated individuals (Bhattacharya et al., 2023[4]). In vitro studies showed that the Delta strain showed approximately 8-fold low susceptibility to vaccine-induced antibodies and neutralizing antibodies in sera from recovered subjects, with higher replication efficiency compared to wild-type Wuhan-1, D614G and the alpha- Variant (Mlcochova et al., 2021[38]). Compared to the other strains, the Delta variant has a different interaction between the RBD and the antibody receptor, making it suitable to evade the immune system and causing infection in vaccinated subjects (Bhattacharya et al., 2023[4]).

Likewise, the Omicron variant complex with over 50 potential mutations and deletions, is capable of infecting and reinfecting the vaccinated individuals. The Omicron variants showed very poor neutralization with the first-generation vaccine and by antibodies derived from pre-Omicron infection (Carabelli et al., 2023[7]). While only bebtelovimab, a monoclonal antibody that targets the RBD of the spike protein, retains efficacy against all SARS-CoV-2 variants. The antibodies generated in response to vaccine or infection do not persist in the immune-system for more than 4 months, hence cannot remain effective when infected by contagious preceeding form of SARS-CoV-2 (Carabelli et al., 2023[7]; Lo Muzio et al., 2021[34]). Due to the short duration of seropositivity for neutralizing antibodies, the possibility of reinfection, raise concerns that vaccination may not confer effective and long-term immunity against SARS-CoV-2 (Lo Muzio et al., 2021[34]). Therefore, booster doses are required to maintain long-term immunity and reduce disease severity. 

During the COVID-19 pandemic, SARS-CoV-2 has demonstrated exemplary ability to accumulate several new mutations in a short period of time that encode new phenotypes and resulted in enhanced transmission, immune escape, or increased pathogenicity. Currently these variants are susceptible to some extent of immunity conferred by infection with native virus strains and against current vaccines (Lo Muzio et al., 2021[34]). With recurrent attenuating mutations, it is speculated that SARS-CoV-2 is now decreasing disease severity after 3 years of the first case, suggesting that SARS-CoV-2 is evolving to become less pathogenic in humans (Carabelli et al., 2023[7]; Cevik et al., 2021[9]; Singh et al., 2021[52]).

## Conclusion

The ability of SARS-CoV-2 to evolve and take over predecessor variants before selective pressure build-up is unrivaled. The coronavirus SARS-CoV-2 strain may evolve into a bit more or less virulent variant depending on mutation appeared. This paradigm of rapid change in the SARS-CoV-2 genome, notably in the RBD of spike glycoprotein indicates the requirement for close monitoring of genomic alteration and integration of genomic data with clinical presentations of mutated SARS-CoV-2. The SARS-CoV-2 evolutionary analysis can help us fight the disease more effectively. The virus will evolve to change its antigenicity and immune response to maintain viability in the host, and it is of vital importance to remain vigilant and continuously monitor the evolving trend of SARS-CoV-2 virus worldwide in order to manage the situation effectively.

## Notes

Pooja Bhardwaj and Shailendra Kumar Mishra contributed equally as first author.

Kamran Zaman and Rajni Kant (Indian Council of Medical Research (ICMR) - Regional Medical Research Center Gorakhpur, BRD Medical College Campus, Gorakhpur-273013, U.P., India; E-mail: rajnikant.srivastava@gmail.com) contributed equally as corresponding author.

## Declaration

### Funding

This research was supported by the Indian Council of Medical Research, New Delhi.

### Acknowledgment

Pooja Bhardwaj expresses gratitude to the HRD, Ministry of Health and Family Welfare, New Delhi, for the DHR Young Scientist fellowship (YSS/2020/000048/PRCYSS). The authors are thankful to Dr Pragya D Yadav and Dr Rima R Sahay for their valuable inputs. 

### Conflict of interest

None.

## Figures and Tables

**Table 1:**
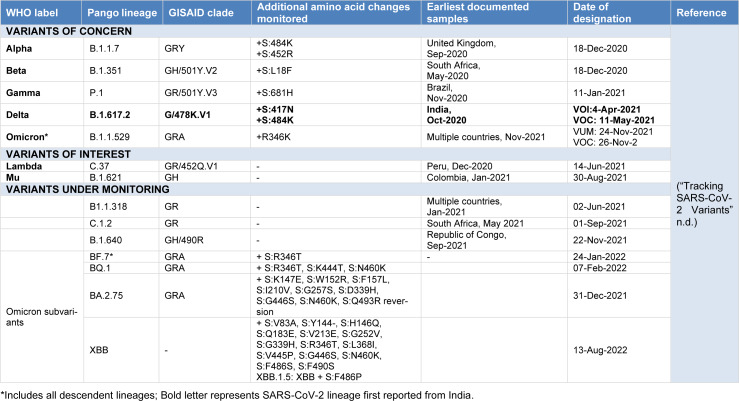
List of SARS-CoV-2 variants currently designated as Variants of Concern, Variants of Interest, and Variants under Monitoring
